# β-Adrenoceptors and synaptic plasticity in the perirhinal cortex

**DOI:** 10.1016/j.neuroscience.2014.04.070

**Published:** 2014-07-25

**Authors:** M. Laing, Z.I. Bashir

**Affiliations:** School of Physiology and Pharmacology, Medical Sciences Building, University Walk, Bristol University, Bristol BS8 1TD, UK

**Keywords:** DMSO, dimethyl sulfoxide, fEPSP, field excitatory postsynaptic potential, LA, lateral amygdala, LTP, long-term potentiation, PRh, perirhinal, β-ADR, β-adrenoceptors, perirhinal cortex, amygdala, β-adrenoceptors, noradrenaline, visual recognition

## Abstract

Experiences with a high degree of emotional salience are better remembered than events that have little emotional context and the amygdala is thought to play an important role in this enhancement of memory. Visual recognition memory relies on synaptic plasticity in the perirhinal cortex but little is known about the mechanisms that may underlie emotional enhancement of this form of memory. There is good evidence that noradrenaline acting via β-adrenoceptors (β-ADRs) can enhance memory consolidation. In the present study we examine the role of β-ADRs in synaptic plasticity at the amygdala–perirhinal pathway (LA–PRh) and compare this to mechanisms of intra-perirhinal (PRh–PRh) synaptic plasticity. We demonstrate that activity-dependent PRh–PRh long-term potentiation (LTP) does not rely on β1- or β2-ADRs and that LA–PRh LTP relies on β1-ADRs but not β2-ADRs. We further demonstrate that application of the β-ADR agonist isoprenaline produces lasting PRh–PRh potentiation but only transient potentiation at the LA–PRh input. However, at the LA–PRh input, combining stimulation that is subthreshold for LTP induction with isoprenaline results in long-lasting potentiation. Isoprenaline-induced and isoprenaline plus subthreshold stimulation-induced potentiation in the PRh–PRh and LA–PRh inputs, respectively were both dependent on activation of NMDARs (N-methyl-D-aspartate receptors), voltage-gated calcium channels and PKA (protein kinase A). Understanding the mechanisms of amygdala–perirhinal cortex plasticity will allow a greater understanding of how emotionally-charged events are remembered.

## Highlights

• Bath application of isoprenaline induces LTP in the perirhinal cortex.
• LTP at amygdala to perirhinal synapses requires β1- but not β2-ADRs.
• Combination of β1-ADRs, NMDARs, and VGCC required for LTP.

## Introduction

The familiarity discrimination component of visual recognition memory requires the perirhinal cortex (Gaffan and Murray, 1992; Fahy et al., 1993; Suzuki et al., 1993; Suzuki, 1996; Ennaceur and Aggleton, 1997; Murray and Bussey, 1999) and synaptic plasticity in the perirhinal cortex may provide the cellular mechanisms underlying recognition memory (Warburton et al., 2003; Griffiths et al., 2008; Massey et al., 2008).

The amygdala has an established role in fear-conditioning paradigms (Blair et al., 2001; Schafe et al., 2001; Maren and Quirk, 2004; Johansen et al., 2010) and is a likely site for the storage of these types of memory (Rogan et al., 1997; Fanselow and LeDoux, 1999; Blair et al., 2001). However, a less well defined yet highly important role of the amygdala is its ability to influence other forms of memory, particularly when the learning episode has a degree of emotional context (Paz et al., 2006).

Experiences with a high degree of emotional context are better remembered than events with less emotional context (McGaugh et al., 2002; Roozendaal et al., 2006, 2009). Application of hormones related to stress and emotions, such as adrenaline or corticosterone, can increase memory consolidation (Roozendaal et al., 2006, 2008, 2009; Barsegyan et al., 2010). The mechanisms by which stress hormones bring about memory consolidation are not well understood but activation of β-adrenoceptors (β-ADR) in the amygdala can enhance object recognition memory (Roozendaal et al., 2008). This suggests a potentially important role of the amygdala in memory consolidation. Activity of the amygdala can regulate cortical activity (Pelletier et al., 2005; Bauer et al., 2007) and can directly influence the spread of neuronal activity from the perirhinal cortex to the entorhinal cortex (Pitkänen et al., 2000; Kajiwara et al., 2003; Paz et al., 2006; Furtak et al., 2007a). These studies suggest that the amygdala is functionally connected with the perirhinal cortex and indeed the amygdala has strong reciprocal connections with the perirhinal cortex (Pitkänen et al., 2000). Taken together, these studies suggest that the amygdala may profoundly influence the mechanisms of recognition memory by direct actions on the perirhinal cortex.

Given that visual recognition memory relies on synaptic plasticity within the perirhinal cortex (Warburton et al., 2003; Griffiths et al., 2008; Massey et al., 2008) it is possible that synaptic transmission or plasticity at the amygdala–perirhinal synapses may be involved in the adrenergic and emotional enhancement of recognition memory. We recently described mechanisms of synaptic plasticity at amygdala–perirhinal synapses and how this input can influence intracortical perirhinal long-term potentiation (LTP) (Perugini et al., 2012). However, little is still known about the role of β-ADRs in the regulation of synaptic plasticity in the perirhinal cortex or in the regulation of amygdala to perirhinal cortex synapses. Therefore, the aim of this study was to further examine in an *in vitro* rat brain slice preparation how synaptic communication at the amygdala–perirhinal cortex synapses and at intracortical perirhinal synapses is influenced by β-ADR activation.

The perirhinal cortex itself is required for visual object recognition (Gaffan and Murray, 1992; Fahy et al., 1993; Suzuki et al., 1993; Suzuki, 1996; Ennaceur and Aggleton, 1997; Murray and Bussey, 1999) and it is known that activation of the noradrenergic system within the amygdala can enhance object recognition memory (Roozendaal et al., 2008).

## Experimental procedures

Juvenile male Lister Hooded rats of 30–35 days of age (70–100 g; Harlan Laboratories, Hillcrest, UK) were maintained on a 12-h light/dark cycle (dark phase during normal daylight). All efforts were made to minimize animal suffering, and experiments were performed in accordance with the UK Animals (Scientific Procedures) Act (1986) and had received ethical approval.

### Slice preparation and electrophysiology

**Fig. 1:**
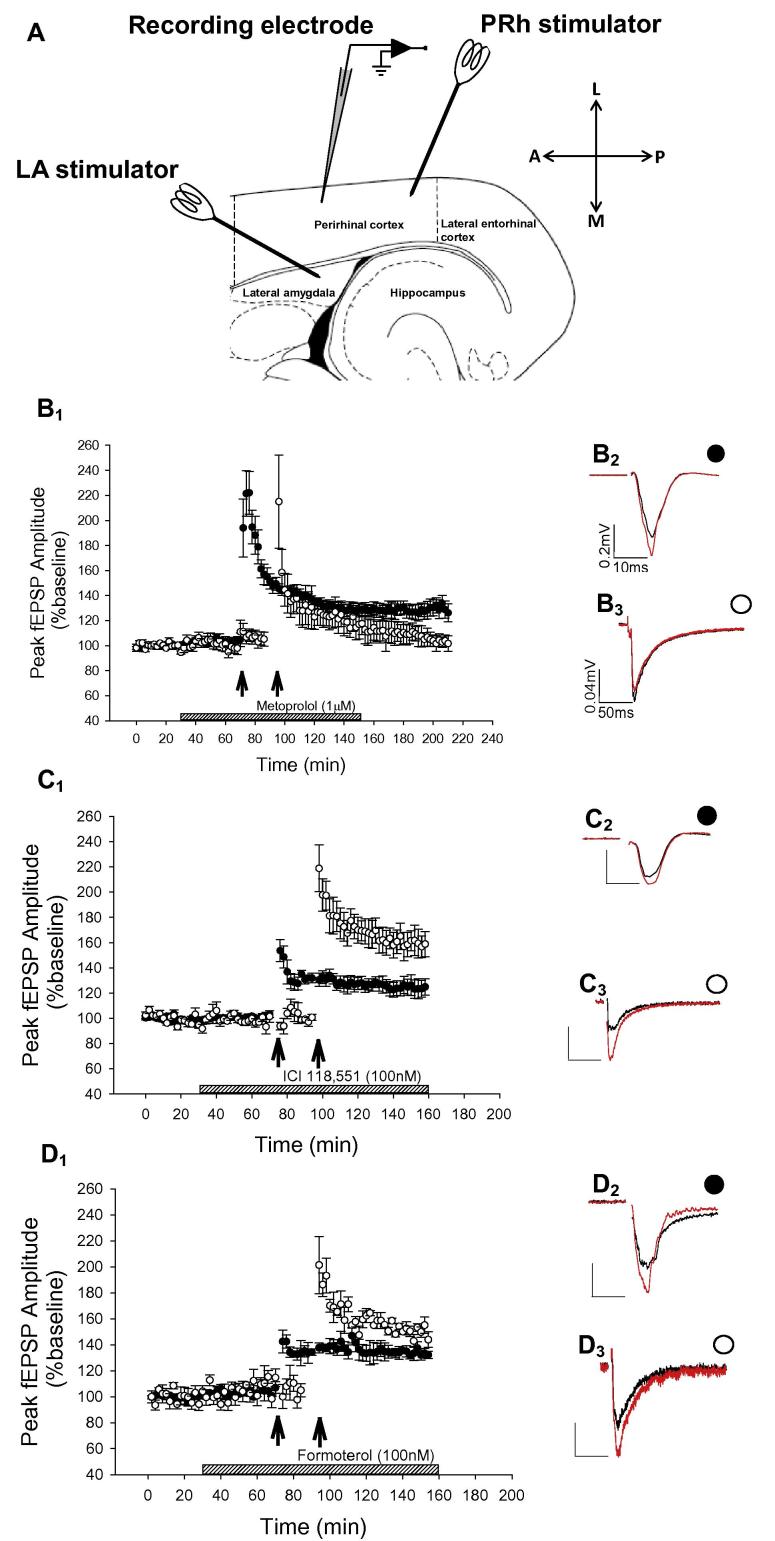
LA–PRh but not PRh–PRh LTP is dependent on β-ADRs. (A) Schematic diagram illustrating the electrode placement in horizontal slices, containing the lateral amygdala and the perirhinal cortex. (B) Metoprolol blocked LA–PRh LTP. (B_1_) Pooled data (*N* = 6) demonstrating that bath application of the β1-specific ADR antagonist metoprolol (1 μM) prevented the induction of LTP in the LA–PRh input (○, *P* = 0.34) but not at the PRh–PRh input (●, *P* = 0.025). (B_2_) Example PRh–PRh traces highlighting the baseline (black) and 1 h after the delivery of HFS (red). (B_3_) Example of LA–PRh traces highlighting the baseline (black) and 1 h after the delivery of HFS (red). (C) ICI 118,551 did not block LTP. (C_1_) Pooled data (PRh–PRh: *N* = 9, LA–PRh: *N* = 10) demonstrating that application of the β2-specific ADR antagonist ICI 118,551 (100 nM) did not block LTP in the LA–PRh (○, *P* ⩽ 0.0001) or in the PRh–PRh (●, *P* = 0.00062) inputs. (C_2_) Example of PRh–PRh traces highlighting the baseline (black) and 1 h after the delivery of HFS (red). (C_3_) Example of traces for the LA–PRh pathway before (black) and after the delivery of the HFS (red). (D) β-ADR agonism did not alter LTP. (D_1_) Pooled data (*N* = 5) demonstrating that bath application of the β2-specific β-ADR agonist formoterol (100 nM) did not alter LTP compared to controls (PRh–PRh *P* = 0.47, LA–PRh *P* = 0.26). (D_2_) Example of PRh–PRh traces highlighting the baseline (black) and 1 h after the delivery of HFS (red). (D_3_) Example of LA–PRh traces highlighting the baseline (black) and 1 h after the delivery of HFS (red). In this and all subsequent figures the values for the x and y axes of scale bars is as in panel (B_2_) and (B_3_). (For interpretation of the references to color in this figure legend, the reader is referred to the web version of this article.)

Animals were anesthetized with an isoflurane/oxygen mixture and decapitated, and the brain rapidly removed. Standard procedures were used for brain slice preparation and *in vitro* extracellular recording. These procedures are detailed fully in Perugini et al. (2012). Briefly, 400-μm thick slices containing perirhinal cortex and lateral nucleus of the amygdala were prepared and stored in standard aCSF (artificial cerebrospinal fluid) at room temperature before use. Single slices were then used for recording at 30–32 °C. Evoked field excitatory postsynaptic potentials (fEPSPs) were recorded with a microelectrode (borosilicate glass micropipette filled with aCSF, 2–5 MΩ) placed in layers II/III of the perirhinal cortex (Massey et al. 2004; Ziakopoulos et al. 1999). Two bipolar stimulation electrodes were placed on the slice: one electrode in layers II/III of the perirhinal cortex, and designated the intracortical input (PRh–PRh). The second stimulating electrode was placed in the lateral amygdala and designated the amygdala input (LA–PRh) (see Fig. 1A). Each input was stimulated at a frequency of 0.033 Hz. In a subset of experiments (Fig. 3D), two stimulating electrodes were placed ∼1.5 mm apart in the lateral amygdala to stimulate amygdala–perirhinal inputs, that were independent of each other. The peak amplitude of fEPSPs was measured and expressed relative to the preconditioning baseline using the LTP program (Anderson & Collingridge, 2001). In all experiments input/output curves were produced by stimulating initially at “minimal” intensity and increased intensities in 3-V steps until the maximal fEPSP was achieved. As can be seen in Fig. 1 the LA–PRh stimulation produces fEPSPs that are very much smaller than with PRh–PRh stimulation and this difference most likely reflects the relatively fewer fibers that reach the PRh cortex from amygdala within the slice preparation. fEPSPs were reduced to 40–50% of the maximum amplitude to achieve a baseline of synaptic transmission. For induction of LTP the following protocols were used: (1) HFS (high frequency stimulation) – four trains of 100 pulses at 100-Hz, 30-s inter-train intervals to either the PRh–PRh or the LA–PRh. (2) for a subset of experiments (Fig. 3) a subthreshold-LTP protocol was applied to the LA–PRh pathway: one train of five pulses at 100-Hz. For all experiments the field potentials were filtered at 5 kHz and digitized at 20 kHz. LTP was measured at 60 min after induction. Changes in response properties were assessed with SigmaPlot 12.0 or GraphPad Prism 6 (where appropriate) using paired or unpaired samples *t* tests or one-way analysis of variances (ANOVAs).

### Pharmacological agents

Compounds were obtained from Tocris Bioscience (Bristol, UK) and prepared as stock solutions (1–10 mM) by dilution in either dimethyl sulfoxide (DMSO) or double-distilled water (ddH_2_O) and stored at −20 °C. Compounds were diluted in aCSF and bath applied at the following concentrations: 20 μM verapamil hydrochloride (ddH_2_O), 15 μM H89 dihydrochloride (ddH_2_O), 200 nM KT 5720 (DMSO), 10 μM nifedipine (DMSO), 10 μM isoprenaline hydrochloride (ddH_2_O), 5 μM L-689,560 (DMSO), 1 μM metoprolol tartrate (ddH_2_O), 100 nM ICI 118,551 hydrochloride (DMSO) and 100 nM formoterol hemifumarate (ddH_2_O).

## Results

### LTP at amygdala to perirhinal synapses requires β1 but not β2-ADRs

We have previously reported that activity-dependent LTP at amygdala–perirhinal (LA–PRh) synapses was prevented by the broad-spectrum β-ADR antagonist propranolol but, in contrast, LTP at perirhinal–perirhinal (PRh–PRh) synapses was not sensitive to propranolol treatment (Perugini et al., 2012). In this study we first sought to examine the subtypes of β-ADRs involved in LTP.

The placement of recording and stimulating electrodes is illustrated in Fig. 1A. Using this placement we firstly confirmed (Perugini et al., 2012) that LTP was induced in both the LA–PRh pathway (148% ± 4%, *t* = 4.5, *P* = 0.004, *N* = 11, data not shown) and in the PRh–PRh pathway (138 ± 6%, *t* = 5.4, *P* = 0.0005, *N* = 11, data not shown). Bath application of the selective β1-ADR antagonist, metoprolol (1 μM) prevented the induction of LA–PRh LTP (103 ± 6%, *t* = 0.44, *P* = 0.34; *N* = 6, Fig. 1B). In contrast, bath application of the selective β2-ADR antagonist ICI 118,551 (100 nM) did not block LA–PRh LTP (166 ± 12%, *t* = 8.7, *P* = 0.0001, *N* = 10, Fig. 1C). LTP was induced at the PRh–PRh input in presence of either the β1-ADR specific antagonist metoprolol (129% ± 7%, *t* = 2.76, *P* = 0.025, *N* = 6, Fig. 1B) or the β2-ADR antagonist ICI 118,551 (129 ± 5%, *t* = 5.2, *P* = 0.00062, *N* = 9, Fig. 1C). The lack of effect of the β1- or β2-ADR antagonists on LTP at the PRh–PRh input was unsurprising, given we have previously demonstrated that the broad-spectrum β-ADR antagonist propranolol did not affect LTP at the PRh–PRh input (Perugini et al., 2012).

While the level of LA–PRh LTP in the presence of the β2-ADR antagonist IC1 118,551 (166 ± 12%) was not significantly greater (*P* = 0.22) than control LTP (148% ± 4%), there was nevertheless a trend toward larger LTP under these conditions (see Fig. 1C). This raises the possibility that under normal conditions there may be some degree of suppression of LTP by endogenous activation of β2-ADRs. Therefore, to examine if this was the case we performed experiments in the presence of the β2-ADR agonist formoterol (100 nM). However, in the presence of formoterol the magnitude of LTP was no different to control LTP in either the PRh–PRh pathway (134 ± 4%, *t* = 3.5, *P* = 0.47, *N* = 5, Fig. 1D) or the LA–PRh pathway (150 ± 4%, *t* = 3.0, *P* = 0.26, *N* = 5, Fig. 1D); this suggests it is unlikely that β2-ADR activation suppresses LTP under the conditions of these experiments.

### Pharmacological activation of β-ADRs at PRh–PRh and LA–PRh synapses

In line with our previous findings (Perugini et al., 2012), the above results demonstrate that LTP induced by HFS at PRh–PRh synapses did not rely on β-ADRs but LTP at LA–PRh synapses did rely on β-ADRs. However, we have previously demonstrated at the PRh–PRh pathway that application of the β-ADR agonist isoprenaline, at a concentration (1 μM) that had no overt effects on synaptic transmission, could promote the induction of LTP by a stimulus protocol that is normally the subthreshold for LTP induction (Perugini et al., 2012).

**Fig. 2:**
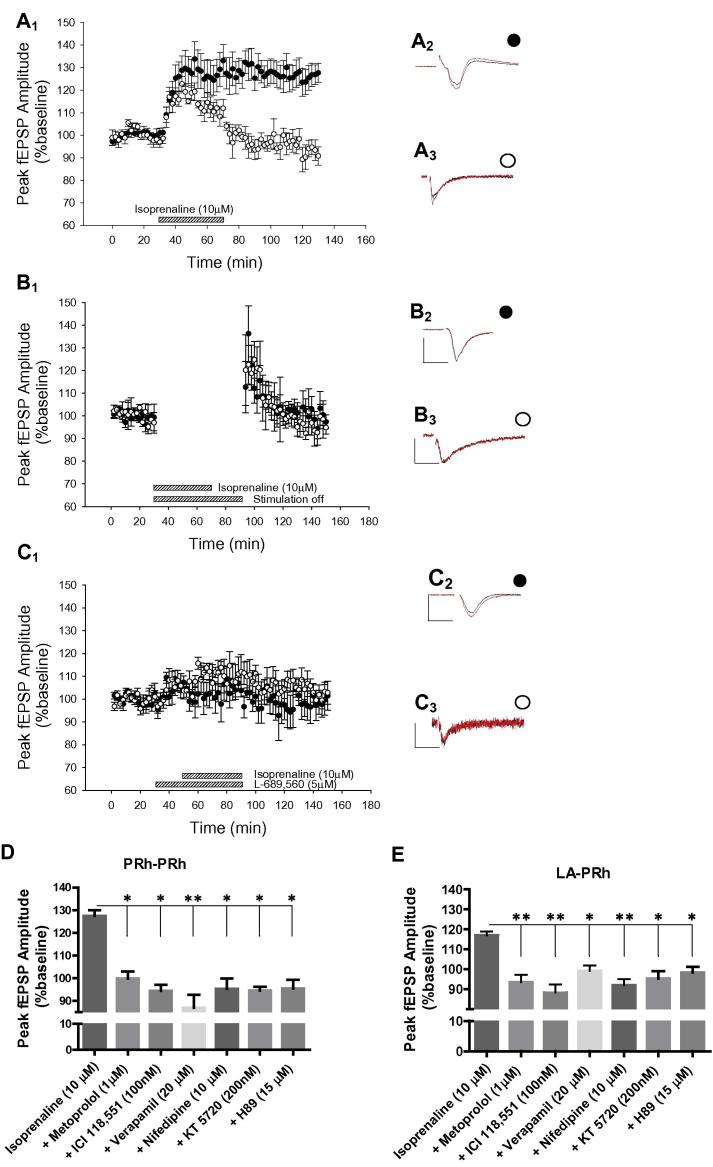
β-ADR activation by isoprenaline induced LTP in the PRh–PRh pathway, that was dependent on NMDA receptors, VGCCs and PKA signaling. (A) Isoprenaline (10 μM) induced LTP in the PRh–PRh pathway but only a transient potentiation in the LA–PRh pathway. (A_1_) Pooled data (*N* = 6) demonstrating the isoprenaline-induced potentiation in the PRh–PRh pathway (●) and the LA–PRh pathway (○). (A_2_) Example of traces for the PRh–PRh pathway, during baseline (black) and 1 h after (red) bath application of isoprenaline. (A_3_) Example of traces for the LA–PRh pathway during baseline (black) and 10–20 min into (red) bath application of isoprenaline. (B) Isoprenaline-induced potentiation of the PRh–PRh input did not occur if stimulation to the slice was ceased during isoprenaline application, and 20 min following washout. (B_1_) Pooled data (*N* = 3) demonstrating that isoprenaline-induced potentiation of the PRh–PRh pathway (●) was prevented (PRh–PRh: *P* = 0.12). (B_2_) Example of traces of the PRh–PRh pathway, during baseline (black) and at the end of the experiment (red). (B_3_) Example of traces for the LA–PRh pathway during baseline (black) and at the end of the experiment (red). (C) Pre-application of the NMDAR antagonist L-689,560 (5 μM) blocked isoprenaline-induced potentiation. (C_1_) Pooled data (*N* = 6) demonstrating that isoprenaline-induced potentiation was blocked in both the PRh–PRh (●, *P* = 0.45) and the LA–PRh inputs (○, *P* = 0.24) when isoprenaline was co-applied with L-689,560. (C_2_) Example of traces of the PRh–PRh pathway, during baseline (black) and at (red) the end of the experiment. (C_3_) Example of traces of the LA–PRh pathway, during baseline (black) and during (red) the co-application of isoprenaline and L-689,560 (10–20 min into co-application). (D) PRh–PRh histogram highlighting that the potentiation observed with the bath application of isoprenaline was blocked by antagonism of β1- or β2-ADRs, VGCCs (verapamil 20 μM or nifedipine 10 μM) or PKA (KT 5720 200 nM or H89 15 μM) or. (E) LA–PRh histogram highlighting that the potentiation observed with the bath application of isoprenaline was blocked by antagonism of β1- or β2-ADRs, VGCCs (verapamil 20 μM or nifedipine 10 μM) or PKA (KT 5720 200 nM or H89 15 μM) Antagonism of either the β1-ADRs (metoprolol) or the β2-ADRs (ICI 118,551) was sufficient to prevent isoprenaline-induced potentiation (measurements taken 10–20 min into co-application). (For interpretation of the references to color in this figure legend, the reader is referred to the web version of this article.)

Therefore to more fully examine the role of β-ADRs in LTP we now performed experiments with bath application of 10 μM isoprenaline. Under these conditions, the application of isoprenaline alone resulted in a potentiation within the PRh–PRh pathway that persisted for at least 1 h after washout (126 ± 6%, *t* = 3.05, *P* = 0.029, *N* = 6, Fig. 2A). Surprisingly however, in the LA–PRh pathway only a transient potentiation was observed (measured 10 min into drug application, 116 ± 3%, *t* = 3.7, *P* = 0.028, *N* = 6, Fig. 2A) and this readily returned to the baseline upon washout of isoprenaline (measured 1 h after washout, 94% ± 4%, *t* = 2.16, *P* = 0.16, *N* = 6, Fig. 2A).

These data, showing that application of isoprenaline resulted in LTP at the PRh–PRh input but not at the LA–PRh input, were surprising given our previous results (Perugini et al., 2012) and the current data demonstrating that β-ADR antagonists block activity-dependent LTP at the LA–PRh pathway but not at PRh–PRh synapses. The isoprenaline result suggests that appropriate activation of β-ADRs has the potential to enhance synaptic transmission in both PRh–PRh and LA–PRh pathways but that very different conditions may be required in the two pathways.

We next examined whether the potentiation induced by bath application of isoprenaline was mediated by β1- or β2-ADRs. Under the conditions of the present experiments we found that the β1-ADR antagonist metoprolol prevented the lasting potentiation induced by isoprenaline in the PRh–PRh input and the transient potentiation in the LA–PRh inputs (PRh–PRh: 99 ± 7%, *P* = 0.56, *N* = 6, Fig. 2D; LA–PRh: 93 ± 9%, *P* = 0.45, *N* = 6, Fig. 2E). In addition, the β2-ADR antagonist ICI 118,551 also prevented potentiation by isoprenaline in both inputs (PRh–PRh: 94 ± 5%, *P* = 0.32, *N* = 4, Fig. 2D; LA–PRh: 88 ± 8%, *P* = 0.34, *N* = 4, Fig. 2E).

### Mechanisms of isoprenaline-induced potentiation

We next tested whether the potentiation by isoprenaline relies upon synaptic stimulation. To achieve this, stimulation was turned off during isoprenaline application and was not resumed until 20 min after isoprenaline had been washed out. Under these conditions no potentiation was observed in either pathway (PRh–PRh; 100% ± 5%, *t* = −0.75, *P* = 0.12, *N* = 3, Fig. 2B; LA–PRh; 96% ± 6%, *t* = −0.88, *P* = 0.22, *N* = 3, Fig. 2B).

That isoprenaline produced potentiation in a stimulus-dependent manner at the PRh–PRh synapses suggested that activation of some other receptor systems are also required for the induction of lasting potentiation. To examine this possibility isoprenaline was applied in the presence of the NMDAR (N-methyl-D-aspartate receptor) antagonist L-689,560 (5 μM); under these conditions application of isoprenaline did not result in LTP in the PRh–PRh pathway (PRh–PRh; 105% ± 3%, *t* = 0.91, *P* = 0.45, *N* = 6, Fig. 2C) and did not produce a transient potentiation in the LA–PRh pathway (LA–PRh; 111 ± 6%, *t* = 0.96, *P* = 0.24, *N* = 6, Fig. 2C). Similarly, isoprenaline-induced lasting potentiation in the PRh–PRh pathway and transient potentiation in the LA–PRh pathway was prevented by either of the voltage-gated calcium channel inhibitors verapamil (PRh–PRh; 88.0% ± 6%, *t* = −0.13, *P* = 0.91, *N* = 5, Fig. 2D; LA–PRh; 97% ± 10%, *t* = −2.20, *P* = 0.160, *N* = 5, Fig. 2E) or nifedipine (PRh–PRh; 100 ± 6%, *t* = −0.46, *P* = 0.68, *N* = 5, Fig. 2D; LA–PRh; 91% ± 4%, *t* = −2.5, *P* = 0.088, *N* = 5, Fig. 2E).

We have previously shown that β-ADR-dependent LTP in the LA–PRh input relied on activation of PKA (protein kinase A) signaling (Perugini et al., 2012). In the current experiments we examined whether the isoprenaline-induced potentiation was also dependent on activation of PKA. We found that the PKA inhibitors KT 5720 (200 nM) or H89 (15 μM) prevented isoprenaline-induced lasting potentiation at PRh–PRh synapses (KT 5720: 99% ± 4%, *t* = −1.7, *P* = 0.15, *N* = 4, Fig. 2D; H89: 108 ± 11%, *t* = −0.095, *P* = 0.93, *N* = 5, Fig. 2D) and prevented the transient potentiation at LA–PRh synapses (KT 5720: 99% ± 8%; *t* = −1.1, *P* = 0.32, *N* = 4, Fig. 2E; H89: 105% ± 5%, *t* = 1.9, *P* = 0.20, *N* = 6, Fig. 2E).

### Lack of occlusion between isoprenaline potentiation and LTP at the PRh–PRh synapses

**Fig. 3:**
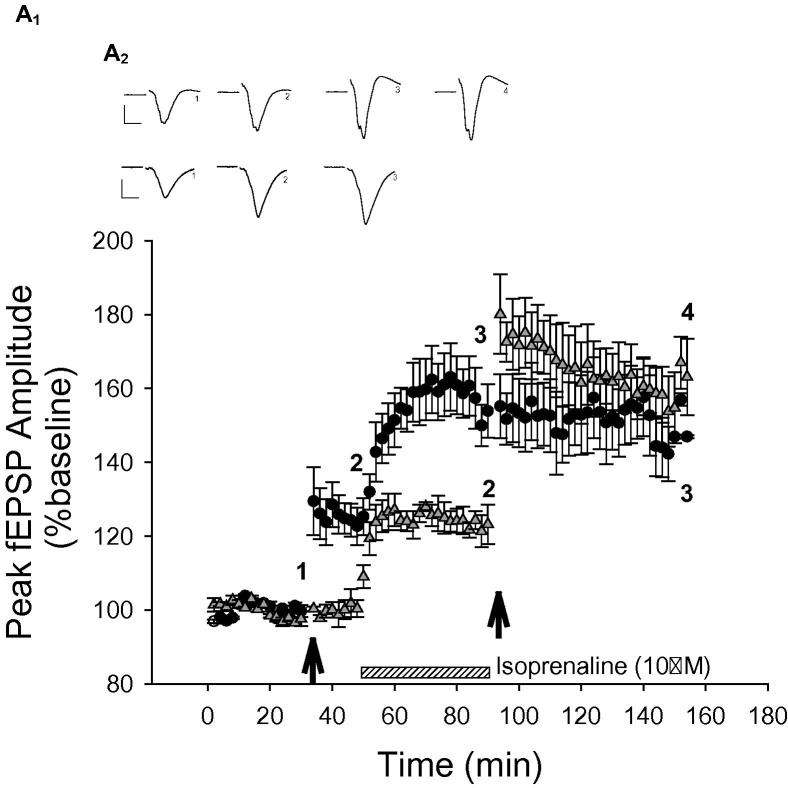
Isoprenaline application did not occlude further potentiation by HFS and vice versa. Example of traces for input 1 (●) of the PRh–PRh response at baseline (1), post-HFS (2), isoprenaline application (3), and at the end of the experiment (4) and for input 2 of the PRh–PRh response at baseline (1), isoprenaline application (2), and the end of the experiment (3).

Two stimulating electrodes were placed intracortically to stimulate separate PRh–PRh inputs. HFS was delivered to input one (*F*_(1,3)_ = 15, *P* = 0.003) to induce LTP (126 ± 6%, *P* = 0.032, *N* = 4, Fig. 3). Isoprenaline was subsequently applied and this resulted in further potentiation in the input in which LTP had already been induced (159 ± 8%, *P* = 0.023, *N* = 4, Fig. 3) and in the input (*F*_(1,2)_ = 17, *P* = 0.004) which had not received HFS (input two, 124 ± 4%, *P* = 0.01, *N* = 4, Fig. 3). After 40 min of isoprenaline application HFS was delivered to the input that had not previously received HFS. This resulted in further sustained potentiation (160 ± 8%, *P* = 0.01, *N* = 4, Fig. 3). Therefore these two forms of potentiation do not occlude one another suggesting that different mechanisms may be responsible for their expression.

### Activation of β-ADRs by isoprenaline lowers the threshold for LTP at the LA–PRh pathway

**Fig. 4:**
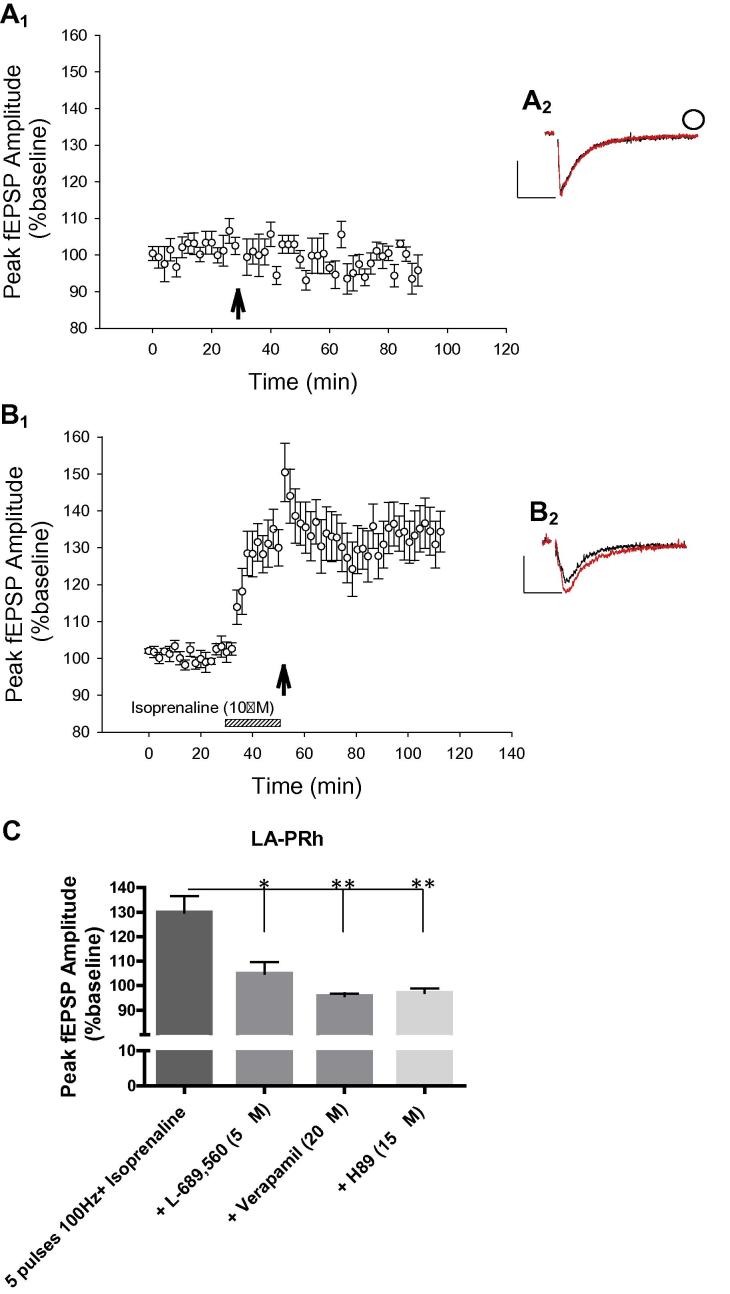
Isoprenaline (10 μM) converted the LA–PRh subthreshold LTP protocol (five pulses 100-Hz) into LTP. (A) Five pulses at 100-Hz delivered alone to the LA–PRh pathway (○), did not significantly potentiate the baseline fEPSP. (A_1_) Pooled data (*N* = 6) demonstrating that five pulses at 100-Hz did not induce significant potentiation in the LA–PRh pathway (○, *P* = 0.25). (A_2_) Example of traces for the LA–PRh pathway before (black) and 1 h after (red) the delivery of five pulses at 100-Hz. (B) Coupling five pulses at 100-Hz with isoprenaline (10 μM), induced LTP at the LA–PRh input. (B_1_) Pooled data (*N* = 6) demonstrating that the subthreshold protocol induced LTP, when coupled with isoprenaline (○, *P* = 0.026). (B_2_) Example of traces for the LA–PRh pathway before (black) and 1 h after (red) the delivery of five pulses at 100-Hz. (C) Summary histogram highlighting that the potentiation induced by isoprenaline, coupled with five pulses at 100-Hz, in the LA–PRh input was blocked by L-689,560 (5 μM, *P* = 0.095), verapamil (20 μM, *P* = 0.24) or H89 (10 μM, *P* = 0.067). (For interpretation of the references to color in this figure legend, the reader is referred to the web version of this article.)

The results so far suggest that activation of β-ADRs may be necessary but not sufficient for LA–PRh LTP. Therefore it is possible that activation of other receptors was also necessary for activity-dependent LA–PRh LTP. To begin to examine this possibility we first identified a protocol which, on its own, was not capable of producing LTP in the LA–PRh pathway (five pulses at 100-Hz; 98 ± 3%, *t* = 0.75, *P* = 0.25, *N* = 6, Fig. 4A). We then coupled this protocol with bath application of isoprenaline (10 μM). Under these conditions, long-lasting potentiation was observed (LA–PRh; 134% ± 6%; *t* = 3.1, *P* = 0.026, *N* = 6, Fig. 4B). We therefore conclude that LA–PRh LTP can be induced by pharmacological activation of β-ADRs in conjunction with activation of some other mechanisms. Therefore, we repeated the above experiment and found that LTP was prevented by the NMDAR antagonist L-689,560 (LA–PRh; 104% ± 11%; *t* = 1.7, *P* = 0.095, *N* = 6, Fig. 4C), the voltage-gated calcium channel inhibitor verapamil (LA–PRh; 96% ± 3%, *t* = −0.81, *P* = 0.24, *N* = 4, Fig. 4C) or the PKA inhibitor H89 (LA–PRh; 96% ± 4%, *t* = −2.4, *P* = 0.067, *N* = 3, Fig. 4C). Therefore LTP in the LA–PRh pathway requires the activation of β-ADRs, NMDARs, voltage-gated calcium channels and PKA signaling, when induced by isoprenaline combined with subthreshold synaptic stimulation.

### Occlusion between isoprenaline potentiation and LTP at the PRh-LA synapses

**Fig. 5:**
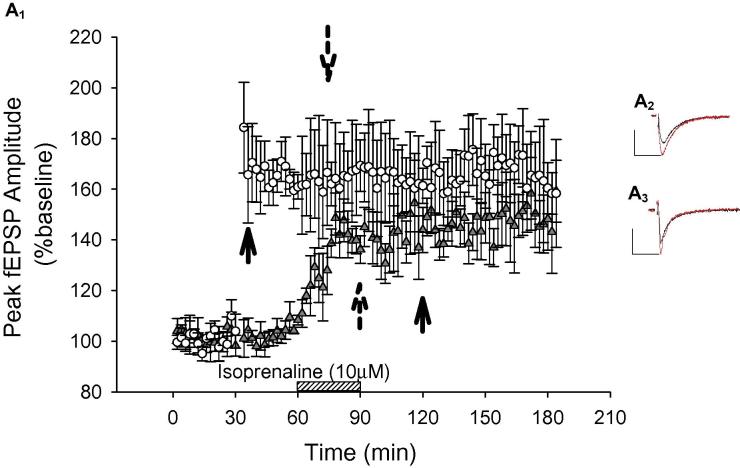
Isoprenaline application occluded further potentiation by HFS, and vice versa. HFS resulted in LTP in input 1 (○) that prevented further potentiation by isoprenaline and subthreshold stimulation (indicated by downward dashed arrow) At input 2, the bath application of isoprenaline, coupled with the subthreshold stimulation (indicated by upward dashed arrow), led to a sustained potentiation. However, subsequent HFS did not further potentiate the input. (A_2_, A_3_) Example of traces for the LA–PRh input during baseline and at the end of the experiment.

Two stimulation electrodes were placed in the lateral amygdala to stimulate independent LA–PRh inputs. HFS was delivered to input one (*F*_(2,9)_ = 5, *P* = 0.031) and this resulted in LTP (164 ± 8%, *P* = 0.033, *N* = 4, Fig. 5). Isoprenaline was then bath applied 30 min after LTP induction. Further potentiation was not induced in this input by the subthreshold induction protocol in the presence of isoprenaline (162 ± 11%, *P* = 0.77, *N* = 4, Fig. 5). In the second input (*F*_(2,9)_ = 9, *P* = 0.007) isoprenaline produced potentiation that was sustained when coupled with subthreshold stimulation (138 ± 8%, *P* = 0.014, *N* = 4, Fig. 5). HFS delivered 30 min later did not produce any further potentiation at this input (147 ± 11%, *P* = 0.082, *N* = 4, Fig. 5). This shows that isoprenaline-facilitated LTP occludes HFS-induced LTP at the LA–PRh input, suggesting similar mechanisms likely underlie both forms of LTP.

**Fig. 6:**
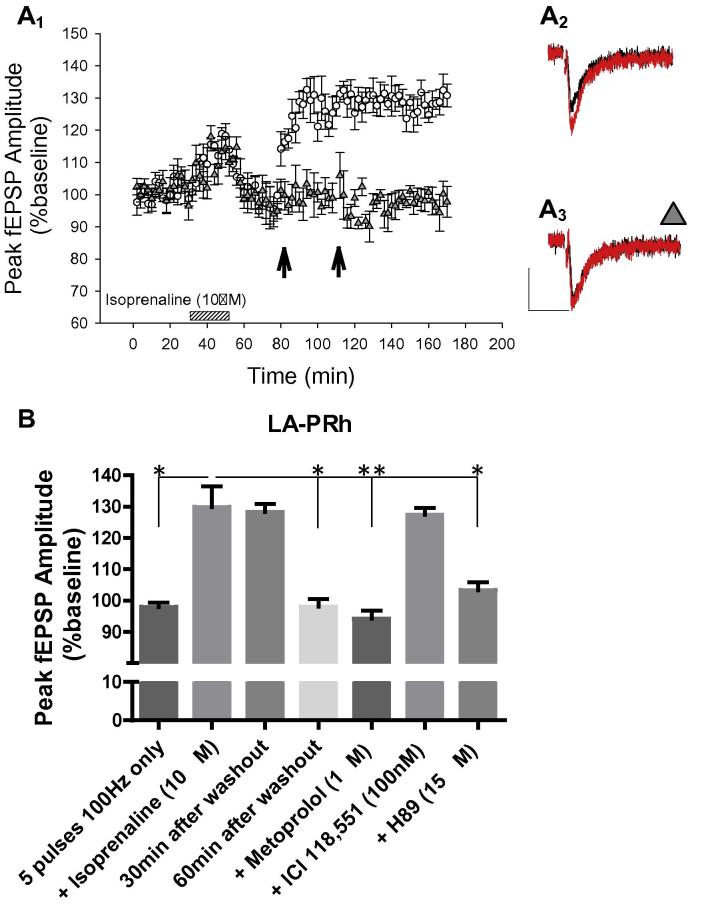
(A) Five pulses at 100-Hz could induce LTP 30 min after isoprenaline washout, but not at 60 min. (A_1_) The subthreshold protocol induced LTP at 30 min after isoprenaline (10 μM) washout in the LA–PRh pathway (Δ, *P* = 0.0065). However, the protocol did not induce LTP 1 h after isoprenaline washout (○, *P* = 0.33). (A_2_) Example of traces for the 30-min delay input for the LA–PRh pathway before (black) and 1 h after (red) delivering five pulses at 100-Hz. (A_3_) Example of traces for the 60-min delay input for the LA–PRh pathway before (in black) and 1 h after (in red) delivering five pulses at 100-Hz. (B) Summary histogram highlighting that LTP induced by subthreshold stimulation and isoprenaline was dependent on β1- or β2ADRs and PKA activation. (For interpretation of the references to color in this figure legend, the reader is referred to the web version of this article.)

In the final set of experiments we investigated whether, to induce LTP, subthreshold HFS needs to be delivered during the transient enhancement of transmission or can be delivered once the transient potentiation has declined back to baseline. Two stimulation electrodes were placed in the lateral amygdala and the subthreshold protocol was delivered at 30 and 60 min following the washout of isoprenaline. LTP was induced when the subthreshold stimulation protocol was delivered 30 min following isoprenaline washout (128% ± 4%, *t* = 5.3, *P* = 0.0065, *N* = 6, Fig. 6A). However, LTP was not induced when the subthreshold stimulation protocol was delivered 60 min after isoprenaline washout (98% ± 4%, *t* = 0.49, *P* = 0.33, *N* = 5, Fig. 6A). To determine whether this sustained potentiation induced by subthreshold stimulation relied on β1- or β2-ADRs we repeated the experiments in the presence of metoprolol or ICI 118,551. LTP was prevented by the β1-ADR antagonist metoprolol (94 ± 5%, *t* = 0.72, *P* = 0.55, *N* = 4, Fig. 6B) but was unaffected by the β2-ADR antagonist ICI 118,551 (127 ± 4%, *t* = 8.4, *P* = 0.001, *N* = 4, Fig. 6B).

The above results suggest that β1-ADR activation triggers an intracellular mechanism that lowers the threshold for LTP and that this intracellular mechanism outlasts the transient synaptic potentiation. However, this mechanism itself is transient and was no longer apparent 60 min after β-ADR activation. This potentiation was blocked by application of H89, delivered 30 min prior to the subthreshold protocol (LA–PRh, 102% ± 4%, *t* = 2.0, *P* = 0.070, *N* = 5, Fig. 6B). Therefore, this transient signaling cascade may be dependent on continued PKA activity triggered by β1-ADR stimulation.

## Discussion

Our previous work demonstrated that activity-dependent LA–PRh LTP was dependent on β-ADRs (Perugini et al., 2012). In the present study we extend our investigation of plasticity at amygdala–perirhinal and perirhinal-perirhinal inputs. We confirm that LTP induced by HFS at the PRh–PRh input is not dependent on β-ADR activation by showing that neither β1- nor β2-ADRs antagonists prevent LTP. We now demonstrate that LTP induced by HFS within the LA–PRh is dependent on β1- but not β2-ADRs. The reason why β1-ADR antagonism blocked, but β2-ADR antagonism did not block, LTP is not clear. Both receptor subtypes are thought to couple to the same Gs protein signaling complex (Gereau and Conn, 1994) and therefore if both receptor subtypes are activated by endogenous noradrenaline one might expect both to be involved in LTP. However, the lack of a role for β2-ADRs might reflect a relatively low level of these receptors in the CNS compared to β1-ADRs (Rainbow et al., 1984; Tiong and Richardson, 1989).

Previous studies have demonstrated an influence of β-ADRs on synaptic plasticity by pairing synaptic stimulation protocols with application of β-ADR agonists (Hopkins and Johnston, 1988; Bröcher et al., 1992; Huang and Kandel, 1996; Thomas et al., 1996; Lin et al., 2003; Choi et al., 2005; Tully et al., 2007). Using a similar approach of pairing stimulation with β-ADR agonist application produced some completely unexpected findings in the current study. Thus, bath application of 10 μM isoprenaline coupled with basal synaptic stimulation resulted in a lasting potentiation at the PRh–PRh input. This was completely surprising given that β-ADRs were not necessary for PRh–PRh LTP induced by HFS. In marked contrast, although β-ADRs were necessary for LTP induced by HFS at the LA–PRh input there was only a transient potentiation induced by 10 μM isoprenaline coupled with basal synaptic stimulation. In previous work we described that 1 μM isoprenaline had no effect on baseline transmission (Perugini et al., 2012), indicating a dose-dependent effect of isoprenaline on the induction of potentiation at PRh–PRh synapses. Previous studies using isoprenaline at 1–50 μM in the CA1 region of the hippocampus and neocortex produced varying results from finding isoprenaline either transiently altered baseline transmission or had no effect (Gelinas and Nguyen, 2005; Seol et al., 2007; Kemp and Manahan-Vaughan, 2008; Salgado et al., 2011).

Importantly these data indicate that although β-ADRs are not necessary for PRh–PRh LTP induced by HFS they are present within this pathway and when appropriately activated (in this case pharmacologically) can induce a lasting potentiation. β-ADRs couple to cAMP and PKA signaling and we have previously shown forskolin application can result in potentiation at the PRh–PRh input (Perugini et al., 2012). Therefore, appropriate activation of cAMP and PKA signaling whether by forskolin or β-ADR activation can contribute to LTP in the PRh–PRh input.

In contrast to the effect at the PRh–PRh input, pharmacological activation of β-ADRs by isoprenaline only produced a transient potentiation at the LA–PRh input when paired with basal stimulation. However, stimulation subthreshold for LTP induction coupled with isoprenaline application did produce LTP at the LA–PRh input. Together, these results suggest that under different circumstances activation of β-ADRs can result in LTP at both the LA–PRh and PRh–PRh inputs. Isoprenaline-induced LTP at both the PRh–PRh and LA–PRh inputs was prevented by either β1- or β2-ADR antagonists. This was in contrast to our finding that LTP induced by synaptic stimulation alone at the LA–PRh input is β1-ADR dependent but not β2-ADR dependent. This therefore suggests that whileβ1- or β2-ADRs may have different expression levels (Rainbow et al., 1984; Tiong and Richardson, 1989), they both have the potential to enhance transmission. Nevertheless the activity-dependent protocols that induce LTP at the LA–PRh input in this study only required β1-ADRs.

An alternative explanation for the differential effects of isoprenaline on synaptic transmission at the PRh–PRh input versus the LA–PRh input is that the threshold for induction of LTP that relies on β-ADR activation is lower at the PRh–PRh input than at the LA–PRh input. This could explain why β-ADR activation by isoprenaline in the PRh–PRh input results in LTP when coupled with basal stimulation at 0.033 Hz but a short burst of 100-Hz stimulation is required to be coupled with isoprenaline stimulation at the LA–PRh input. The different stimulation protocols will most likely produce different levels of NMDA receptor and L-type VGCC activation with lower levels being required at the PRh–PRh input compared to the LA–PRh input.

Interestingly, HFS-induced LTP and isoprenaline-induced potentiation in the PRh–PRh input did not occlude one another, suggesting that different mec-hanisms may underlie these forms of lasting potentiation. However, our previous data (Perugini et al., 2012) and the current data show that similar intracellular mechanisms most likely operate in both these forms of potentiation. Thus it is possible that the final expression mechanisms of potentiation are different. In contrast, HFS-induced LTP did occlude isoprenaline plus subthreshold stimulation-induced potentiation (and vice versa) at the LA–PRh input. This suggests that the intracellular mechanisms and/or the expression mechanisms of both forms of potentiation at the LA–PRh input are likely to be similar.

Since the isoprenaline-induced potentiation in both the PRh–PRh and the LAPRh inputs was stimulus-dependent we hypothesized that- this potentiatio-n may rely on glutamate release and activation of glutamate receptors. Induction of synaptic plasticity requiring subthreshold stimulation and isoprenaline application has also been reported previously (Kirkwood et al., 1999; Seol et al., 2007). We found that blockade of NMDARs with L-689,560 prevented the isoprenaline-induced potentiation. The reason for the combined role of β-ADRs and NMDARs in this form of LTP is not clear but enhancement of neuronal excitability by β-ADR activation (Heginbotham and Dunwiddie, 1991; Pedarzani and Storm, 1995) could promote NMDAR activity and thus promote LTP induction. Alternatively, there is evidence for a functional link between β-ADRs and NMDARs as part of a larger AMPAR, AKAP, β-ADR and NMDAR signaling complex located at the plasma membrane (Colledge et al., 2000; O’Dell et al., 2010). In addition, a direct interaction between AMPARs and β-ADRs receptors has been postulated to result in the phosphorylation of the GluR1 AMPAR subunit and an increase in surface AMPARs (Joiner et al., 2010) which may underlie either or both transient potentiation and LTP. Finally, it is possible that β-ADR activation enhances downstream cascades triggered by NMDAR activation (Seol et al., 2007). In addition, both transient potentiation at the LA–PRh input and sustained potentiation at the PRh–PRh input were also prevented by blockade of voltage-gated calcium channels and inhibition of PKA. β-ADR activation has been previously linked to L-type calcium channels and the mechanism involves PKA-mediated phosphorylation of the L-type calcium channels to increase channel conductance (Catterall, 2000). This suggests that β-ADRs stimulated PKA, NMDARs and calcium channel activity can all contribute to both the lasting potentiation at the PRh–PRh and the transient potentiation at the LA–PRh inputs.

In the final part of this study we were able to demonstrate at the LA–PRh input that stimulation subthreshold for induction of LTP could induce LTP when delivered 30 min after washout of isoprenaline, at a time when there was no transient potentiation. This result is similar to previous findings in the visual cortex (Seol et al., 2007) and hippocampus (Gelinas et al., 2008) and suggests that isoprenaline application produces some lasting change in synaptic function (that outlasts the transient potentiation) that can enhance synaptic plasticity. Interestingly this form of LTP was blocked by β1-ADR antagonism but not β2-ADR antagonism and therefore parallels HFS-induced LTP at this input. The exact mechanisms underlying this prolonged excitability remain unknown. However, in our experiments blocking PKA activity prevented the induction of this form of LTP. This agrees with a previous study (Gelinas et al., 2008) suggesting that the mechanism of enhancement of plasticity within the LA–PRh pathway may rely on constitutive activity of PKA.

In conclusion, this study increases understanding of mechanisms of synaptic plasticity within the perirhinal cortex resulting from intracortical stimulation and also following activity of the lateral amygdala output to perirhinal cortex. These mechanisms may be important for visual recognition memory and for modulation of such memory by emotional experience.

## Contributions

M.L. conducted the experiments, helped to develop the study, and contributed to the writing of the paper.

Z.I.B. conceived and supervized the study and wrote the paper.

Funded by Medical Research Council (UK) PhD studentship to M.L.
